# Transcriptomic and metabolomic correlation analysis: effect of initial SO_2_ addition on higher alcohol synthesis in *Saccharomyces cerevisiae* and identification of key regulatory genes

**DOI:** 10.3389/fmicb.2024.1394880

**Published:** 2024-05-13

**Authors:** Yuan Lin, Na Zhang, Yonghong Lin, Yinhao Gao, Hongxing Li, Cuixia Zhou, Wu Meng, Weishuai Qin

**Affiliations:** ^1^State Key Laboratory of Biobased Material and Green Papermaking (LBMP), Qilu University of Technology (Shandong Academy of Sciences), Jinan, China; ^2^College of Biology and Brewing Engineering, Taishan University, Taian, China

**Keywords:** higher alcohols, transcriptomics, metabolomics, SO2 addition, *Saccharomyces cerevisiae*

## Abstract

**Introduction:**

Higher alcohols are volatile compounds produced during alcoholic fermentation that affect the quality and safety of the final product. This study used a correlation analysis of transcriptomics and metabolomics to study the impact of the initial addition of SO_2_ (30, 60, and 90 mg/L) on the synthesis of higher alcohols in *Saccharomyces cerevisiae* EC1118a and to identify key genes and metabolic pathways involved in their metabolism.

**Methods:**

Transcriptomics and metabolomics correlation analyses were performed and differentially expressed genes (DEGs) and differential metabolites were identified. Single-gene knockouts for targeting genes of important pathways were generated to study the roles of key genes involved in the regulation of higher alcohol production.

**Results:**

We found that, as the SO_2_ concentration increased, the production of total higher alcohols showed an overall trend of first increasing and then decreasing. Multi-omics correlation analysis revealed that the addition of SO_2_ affected carbon metabolism (ko01200), pyruvate metabolism (ko00620), glycolysis/gluconeogenesis (ko00010), the pentose phosphate pathway (ko00030), and other metabolic pathways, thereby changing the precursor substances. The availability of SO_2_ indirectly affects the formation of higher alcohols. In addition, excessive SO_2_ affected the growth of the strain, leading to the emergence of a lag phase. We screened the ten most likely genes and constructed recombinant strains to evaluate the impact of each gene on the formation of higher alcohols. The results showed that *ADH4, SER33*, and *GDH2* are important genes of alcohol metabolism in *S. cerevisiae*. The isoamyl alcohol content of the EC1118a*-ADH4* strain decreased by 21.003%; The isobutanol content of the EC1118a*-SER33* strain was reduced by 71.346%; and the 2-phenylethanol content of EC1118a*-GDH2* strain was reduced by 25.198%.

**Conclusion:**

This study lays a theoretical foundation for investigating the mechanism of initial addition of SO_2_ in the synthesis of higher alcohols in *S. cerevisiae*, uncovering DEGs and key metabolic pathways related to the synthesis of higher alcohols, and provides guidance for regulating these mechanisms.

## 1 Introduction

In vinification, sulfur dioxide (SO_2_) is the most common, cheap, and effective food additive. SO_2_ has multiple beneficial effects, including antibacterial and antioxidant properties and a satisfactory impact on wine color (Lisanti et al., 2019; Capece et al., 2020). Once added to must or wine, total SO_2_ consists of various bound and free forms of the compound in equilibrium, mainly molecular SO_2_, bisulfite, and sulfite (Sacks et al., 2020). However, ingestion of SO_2_ can cause adverse health effects, such as diarrhea, abdominal pain, and urticaria, and excessive intake and cumulative consumption of SO_2_ may lead to vital organ poisoning (Vally et al., 2009; Ferrer-Gallego et al., 2017). Excess SO_2_ is toxic to yeast, and its initial level will affect the duration of the lag phase and the production of lipids, phenols, alcohols and other substances (Ferreira et al., 2017). Although many SO_2_ alternatives have shown good efficacy, no other physical technology or additive currently offers the same efficacy and broad spectrum of advantageous effects (antioxidant and antimicrobial) as SO_2_. Therefore, alternative methods should be considered as supplements to SO_2_ in low-sulfite winemaking rather than as a complete replacement (Lisanti et al., 2019).

**Figure 1:**
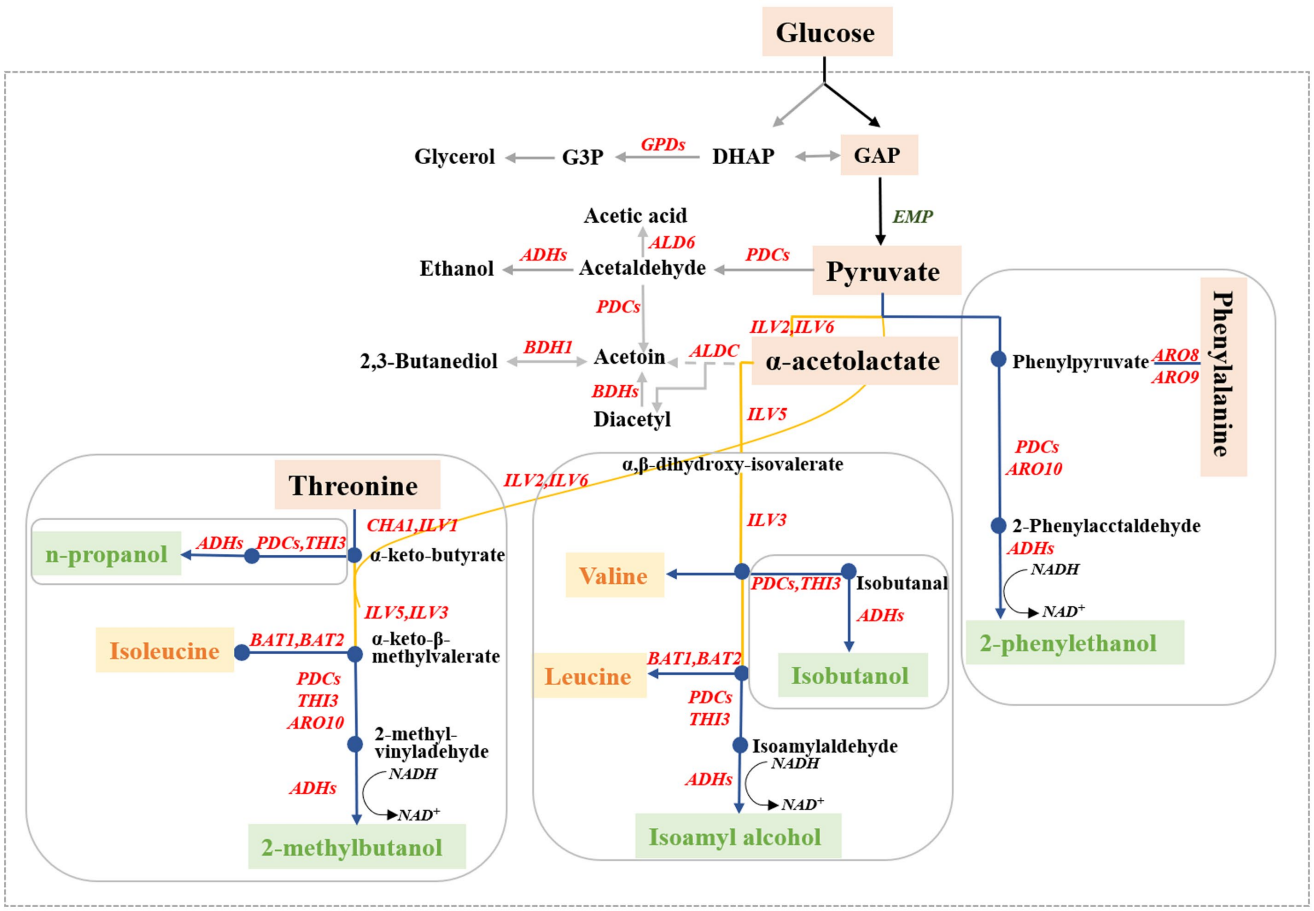
Biosynthetic pathways for higher alcohol formation in *Saccharomyces cerevisiae*. The blue line represents the higher alcohols originating from the Ehrlich pathway, the yellow line represents the synthesis of the corresponding amino acids, and the gray line represents the higher alcohol-related metabolites.

Higher alcohols, commonly known as fuel oils, are important volatile flavor compounds in alcoholic beverages and significantly affect the odor and texture of wine (Li et al., 2018). The main higher alcohols produced by *S. cerevisiae* are n-propanol, isobutanol, isoamyl alcohol, and 2-phenylethanol (Choi et al., 2014). Excessive amounts of higher alcohols have strong intoxicating properties and negatively impact the quality (off-flavor), health effects, (headaches), and safety (poisoning) of alcoholic beverages (Yang et al., 2014; Sun et al., 2020). In *S. cerevisiae*, higher alcohols are generated through decarboxylation reduction of the corresponding intermediate α-keto acid to its corresponding aldehyde, followed by a subsequent dehydrogenation reaction (Cordente et al., 2021). The formation of higher alcohols in yeast can be divided into two different pathways based on the origin of the intermediate α-keto acids (Figure 1). One pathway, called the Ehrlich pathway, involves the catabolic generation of α-keto acids through the amino acid transamination pathway; the other pathway, called the Harris pathway, involves the biosynthetic generation of α-keto acids through glucose metabolism and the tricarboxylic acid cycle (Wang et al., 2017).

Based on transcriptomic analysis, the key genes that affect higher alcohols under different temperatures and α-amino nitrogen concentrations have been revealed (Sun et al., 2019; Wang et al., 2021). The effect of SO_2_ addition on volatile substances such as aldehydes, lipids, and alcohols during the wine fermentation process has also been explored (Herrero et al., 2003; Ochando et al., 2020). However, few studies have used transcriptomic and metabolomic correlation analyses to explore the effect of initial SO_2_ addition on higher alcohol synthesis in *S. cerevisiae* during winemaking or to explore the key genes involved.

The integration of multi-omics approaches will complement existing molecular and genetic information and provide valuable insights into the molecular mechanisms involved (Yu et al., 2022). Transcriptomics and metabolic phenotyping analysis enable us to gain a deep understanding of the functions of differentially expressed genes (DEGs), metabolic pathways, and regulatory mechanisms involved in the synthesis of higher alcohols (He et al., 2023). By employing strategies such as deletion and overexpression to alter the transcription levels of key genes in this pathway, we identified and verified the key genes involved in the synthesis of higher alcohol and their competition pathways in *S. cerevisiae*, thereby controlling the content of higher alcohols produced (Cui et al., 2023).

This study investigated the effect of the initial addition of SO_2_ to the fermentation system on the production of higher alcohols in yeast through transcriptomics and metabolomics correlation analysis and screened the DEGs that are identified by further analysis. Constructing a single-gene knockout recombinant strain, targeting important pathways, uncovering intrinsic patterns, and mining DEGs to identify key genes and metabolic pathways that regulate higher alcohol metabolism will provide crucial guidance for the precise regulation of higher alcohol production.

## 2 Materials and methods

### 2.1 Strains, plasmids, and primers

*Saccharomyces cerevisiae* EC1118a used in this study was provided and preserved by the State Key Laboratory of Microbiology, School of Bioengineering, Qilu University of Technology. The plasmids and primers used in this study are listed in Supplementary Tables S1, S2, respectively. Enzyme preparations used in this study were purchased from Vazyme Biotechnology Co., Ltd. (Nanjing, China).

### 2.2 Culture medium and experimental design

*Saccharomyces cerevisiae* was cultured in YPD medium (containing 1% yeast extract, 2% peptone, and 2% glucose); 200 μg/mL of G418 (geneticin, Coolaber, Beijing, China) was added to YPD medium to screen for KanMX-resistant transformants in recombinant strains. YPG medium (1% yeast extract, 2% peptone, and 2% galactose) was used to select strains with KanMX resistance gene eliminated. The grape juice culture medium was prepared from Chardonnay grapes that were destemmed, crushed, and pressed to extract the juice. After centrifugation, the juice was sterilized and set aside for later use (total sugar, 206 g/L; total acid: 5.8 g/L; pH 3.6). SO_2_ (in the form of K_2_S_2_O_5_) at 30, 60, and 90 mg/L was added to the grape juice culture medium. Each concentration has three parallel groups, and equal amounts of yeast cells were inoculated into grape juice culture medium. At the end of fermentation, the concentrations of isobutanol, isoamyl alcohol, and 2-phenylethyl alcohol were determined. A UV spectrophotometer was used to measure optical density at 600 nm to determine the biomass concentration and draw a standard curve.

### 2.3 Total RNA extraction and quality assessment

Yeast cells cultured in grape juice medium containing various concentrations of SO_2_ were harvested during the logarithmic phase of growth, centrifuged at a low temperature (8,000 × g, 4°C), and immediately frozen in liquid nitrogen. Total RNA was extracted using the TRIzol reagent. The quality and integrity of RNA nucleic acid samples were checked using agarose gel electrophoresis and Agilent 2,100 Bioanalyzer (Agilent Technologies Inc., CA, United States). Measuring the purity and concentration of RNA nucleic acid samples using the NanoDrop™ One/OneCsystem (Thermo Fisher Scientific, MA, United States).

### 2.4 Transcriptome library construction and sequencing analysis

After enriching the eukaryotic mRNA with a poly A tail using magnetic beads with oligo (dT), the mRNA is fragmented using ultrasound and reverse transcribed into the first strand of cDNA (Cui et al., 2023). The RNA chain was degraded with RNase H, and the second strand of cDNA was synthesized using dNTPs as the raw material in the DNA polymerase I system. Purified double-stranded cDNA was end-repaired, A-tailed, and connected to sequencing adapters (Peng et al., 2023). AMPure XP beads were used to screen approximately 200 bp of cDNA. PCR amplification was performed. Finally, AMPure XP beads were used to purify the PCR products and obtain a library (Duan et al., 2022). The sequencing strategy was PE150, and the sequencing platform was Illumina Novaseq 6,000. Sequencing of cDNA libraries was done using an Illumina sequencing platform by GeneDenovo Biotechnology Co., Ltd. (Guangzhou, China).

Use Fastp (v0.18.0) to perform quality control on the raw reads off the machine, and filter low-quality data to obtain clean reads. Use the short read alignment tool Bowtie2 (v2.2.8) to align the clean reads to the ribosomal RNA database, remove the aligned ribosomal reads without allowing mismatches, and use the remaining unmapped reads for subsequent transcriptomes analyze. HISAT2 (v2.2.4) (Kim et al., 2015) was used to align the sequences obtained by paired-end sequencing to the reference genome (Ensembl_release47).^1^ Stringtie was used to reconstruct transcripts based on the comparison results, and RSEM (v1.3.1) was used to quantify expression abundance and variation through FPKM values (number of transcript fragments per kilobase/million mapped reads).we used R language gmodels (v2.18.1)^2^ to carry out principal component analysis (PCA) to study the distance relationship between samples through dimensionality reduction. Differential gene expression analysis was performed using the DESeq2 (Love et al., 2014) software. Genes meeting the criteria FDR1 < 0.05 and |log2FC| > 1 were considered as significant DEGs.

### 2.5 Metabolomics experimental method

After slowly thawing 6 sets of parallel samples and quality control samples (QC Dunn et al., 2011) at 4°C, take an appropriate amount of samples and add them to the pre-cooled solution (methanol: acetonitrile: aqueous solution = 2:2:1, v/v) Mix well, sonicate at low temperature for 30 min, then let stand at −20°C for 10 min, and centrifuge at low temperature for 20 min (14,000 × g, 4°C). Take the supernatant and dry it under vacuum. During mass spectrometry analysis, add 100 μL acetonitrile aqueous solution (acetonitrile: aqueous solution = 1:1, v/v) to reconstitute. After centrifugation for 15 min (14,000 × g, 4°C), the supernatant was taken as the loading solution. Equal volumes from the samples were used to determine the instrument status and equilibrium chromatography prior to injection. Mass spectrometry was used to evaluate system stability during the experimental process. LC–MS/MS (Triple quadrupole: Q-TOF) analysis was performed to quantify and characterize metabolites, followed by data quality evaluation and analysis.

### 2.6 Metabolome quality control and analysis

Preprocess the original data and use ProteoWizard software to convert the original data into. MzML format. The XCMS program was then used to extract the data and perform data integrity checks and normalization of the total peak area of the data to ensure parallel comparisons between samples and metabolites. Two ionization methods, positive ion mode (POS) and negative ion mode (NEG), are used for data quality control, and the data can be analyzed with higher coverage and better detection results. The R language pheatmap (Wu et al., 2022) was used to standardize the data *z*-score and perform cluster analysis, combined with the VI *p* value of the multivariate statistical analysis PLS-DA (Bylesjö et al., 2006) and the univariate statistical analysis *T*-test *p* value to screen for significantly different metabolites between different comparison groups (Saccenti et al., 2014). Utilizing the rich pathway data from differential metabolites, we constructed a metabolic relationship network diagram to elucidate the interactions between different pathways, aiding in the identification of core metabolic pathways. The threshold for significant differences was VIP ≥ 1 and a *t*-test <0.05 in the PLS-DA model.

### 2.7 Enrichment analysis of differentially expressed genes and metabolites

The Gene Ontology (GO) database^3^ was used for the GO functional analysis. GO functional classification annotations of DEGs help determine the functions of these genes, while GO functional significance enrichment analysis determines the main biological functions of DEGs. The Kyoto Encyclopedia of Genes and Genomes (KEGG)^4^ was used to analyze large-scale molecular datasets generated from molecular-level information, especially through high-throughput experimental techniques such as genome sequencing, to understand the advanced functions and utility of biological systems (Wang et al., 2021).

### 2.8 Transcriptomics and metabolomics correlation analysis

To screen and obtain a set of correlated genes and metabolites that influence sample grouping and analyze correlation characteristics, three model analyses were performed based on gene expression and metabolite abundance data. First, a pathway functional model was used to query the KEGG metabolic pathways shared by genes and metabolites and analyze their correlation characteristics (Cho et al., 2016). Second, gene expression and metabolite abundance data were used to construct a bidirectional orthogonal projections to latent structures (O2PLS) model (el Bouhaddani et al., 2016); through model prediction, we obtained a combined analysis of related gene and metabolite sets. Finally, when the sample group was ≥3, a correlation coefficient model was used to calculate the Pearson correlation coefficient between gene expression and metabolite abundance (Bartel et al., 2015; Hamanishi et al., 2015). The model analysis was visualized as a heat map and network diagram.

### 2.9 Construction of recombinant strains and determination of higher alcohol production

Using the yeast genome as a template, the upstream and downstream homologous fragments of the target gene were PCR amplified. Additionally, the plasmid pUG6 was used as a template to PCR amplify the Loxp-KanMX-Loxp resistance fragment to construct the knockout component. These three knockout component fragments were transformed into the parental strain using the lithium acetate/polyethylene glycol (PEG) method (Gietz and Schiestl, 2007) to achieve homologous recombination between the transformed fragments and the yeast genome (Supplementary Figure 1). Transformants were initially screened on YPD agar containing 200 μg/mL G418, and PCR was used to confirm the accurate integration of the KanMX knockout cassette from a single colony, which was subjected to two strain activations. Subsequently, lithium acetate/ PEG method was used to transform the plasmid pSH69 into the abovementioned colonies containing the correct integration. Plasmid transformation was verified. Colonies containing the plasmids were induced and cultured on YPG agar medium. KanMX-resistant fragments were removed through the expression of Cre recombinase induced by galactose in the medium. Subsequently, the plasmid pSH69 was lost through subculture, and the recombinant strain with the desired knockout was obtained.

A GC–MS method was used to determine the content of higher alcohols. An 8 mL sample was transferred into a 15 mL sample bottle with 1.5 g NaCl and 100 μL butyl acetate (internal standard) and preheated to 45°C for 10 min using a magnetic stirrer/heating stage. The material was then extracted with a DVA/CAR/PDMS extraction head, and analyzed using GC–MS. Each sample was analyzed three times. This was done on an InertCap capillary column (30 mm × 0.25 mm × 0.25 μm) using the temperature protocol: hold at 40°C for 3 min, heat to 130°C at 2°C/min, and heat to 220°C at 10°C/min retention for 4 min; the injector and detector temperature 250°C, and no split injection; electron impact ion source, electron energy 70 eV, ion source temperature 230°C, and full scan mode. Based on the chromatographic retention time and mass spectrometry information of the standard product, quantitative analysis was performed by comparing the qualitative and internal standard methods with reference to the standard spectral library (NIST17).

### 2.10 Reverse transcriptase quantitative PCR

To perform fluorescence reverse transcriptase real-time quantitative PCR (qPCR), RNA was extracted using the SPARK Easy Yeast RNA, and Script II RT Plus Kits (Sparkjade Biotechnology Co., Ltd., Shandong, China) was used to reverse transcribe the RNA. Taq Pro Universal SYBR qPCR Master Mix was obtained from Vazyme Biotechnology (Nanjing, China). The PCR procedure consisted of an initial pre-denaturation at 95°C for 30 s, followed by amplification consisting of 40 cycles of denaturation at 95°C for 5 s, annealing and polymerization at 60°C for 30 s, and a melting curve stage at 95°C for 15 s and at 60°C for 1 min. The ubiquitin-conjugating enzyme E26 gene *UBC6* was used as the internal reference gene, and the 2^−ΔΔCt^ method was used for quantitative analysis (Teste et al., 2009).

### 2.11 Statistical analysis

All experiments were conducted in at least three parallel groups, and statistical analysis was performed in GraphPad Prism 9.5.1 (San Diego, California, United States). The results were expressed as mean and standard deviation (SD), and were analyzed using one-way analysis of variance (ANOVA). There was a significant difference, and the statistical significance level was set at *p* value <0.05.

## 3 Results

### 3.1 Effect of initial SO_2_ addition on higher alcohol synthesis and growth of the EC1118a strain

**Figure 2:**
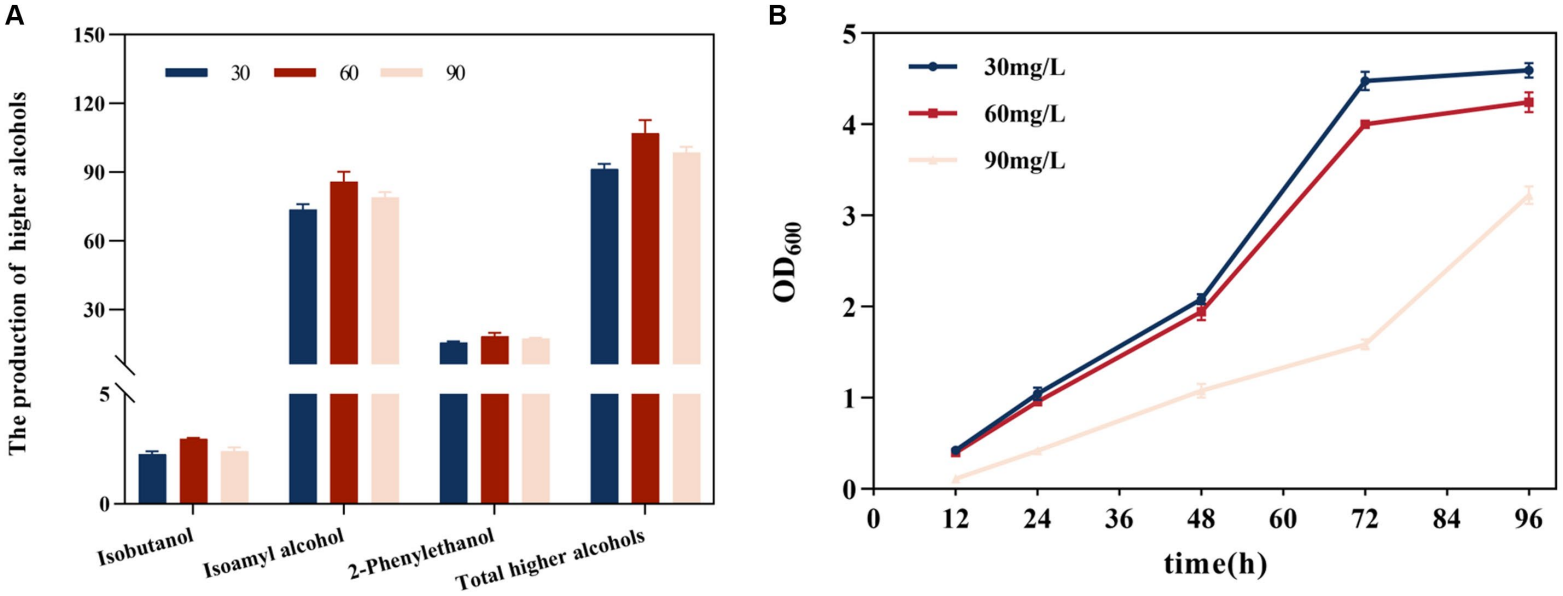
Effect of the initial total SO_2_ concentration on the formation of higher alcohols. **(A)** Effect on isobutanol, isoamyl alcohol, 2-phenylethanol, and the cumulative concentration of higher alcohols. **(B)** Effect on yeast strain growth. The data are the mean ± SD from three biological replicates.

The generation of higher alcohols was correlated with SO_2_ concentration (Figure 2A). At 30, 60, and 90 mg/L SO_2_, the total alcohol content was 91.513, 113.677, and 98.649 mg/L, respectively. Compared with the 30 mg/L SO_2_, total high alcohol content increased by 16.892 and 7.797% at the 60 and 90 mg/L SO_2_, respectively; this involved an increase in the amounts of isobutanol by 31.064 and 5.964%, isoamyl alcohol by 16.473 and 7.163%, and 2-phenylethyl alcohol by 16.824 and 11.064%, respectively. The yield of higher alcohols showed an overall trend of being highest at 60 mg/L SO_2_, and showed a decline at 90 mg/L SO_2_. As the initial SO_2_ concentration increased, yeast growth was inhibited (Figure 2B). The inhibitory effect was evident at 90 mg/L, and the duration of the lag phase was significantly prolonged with increasing SO_2_ concentrations.

### 3.2 Transcriptome analysis and screening of DEGs

**Figure 3:**
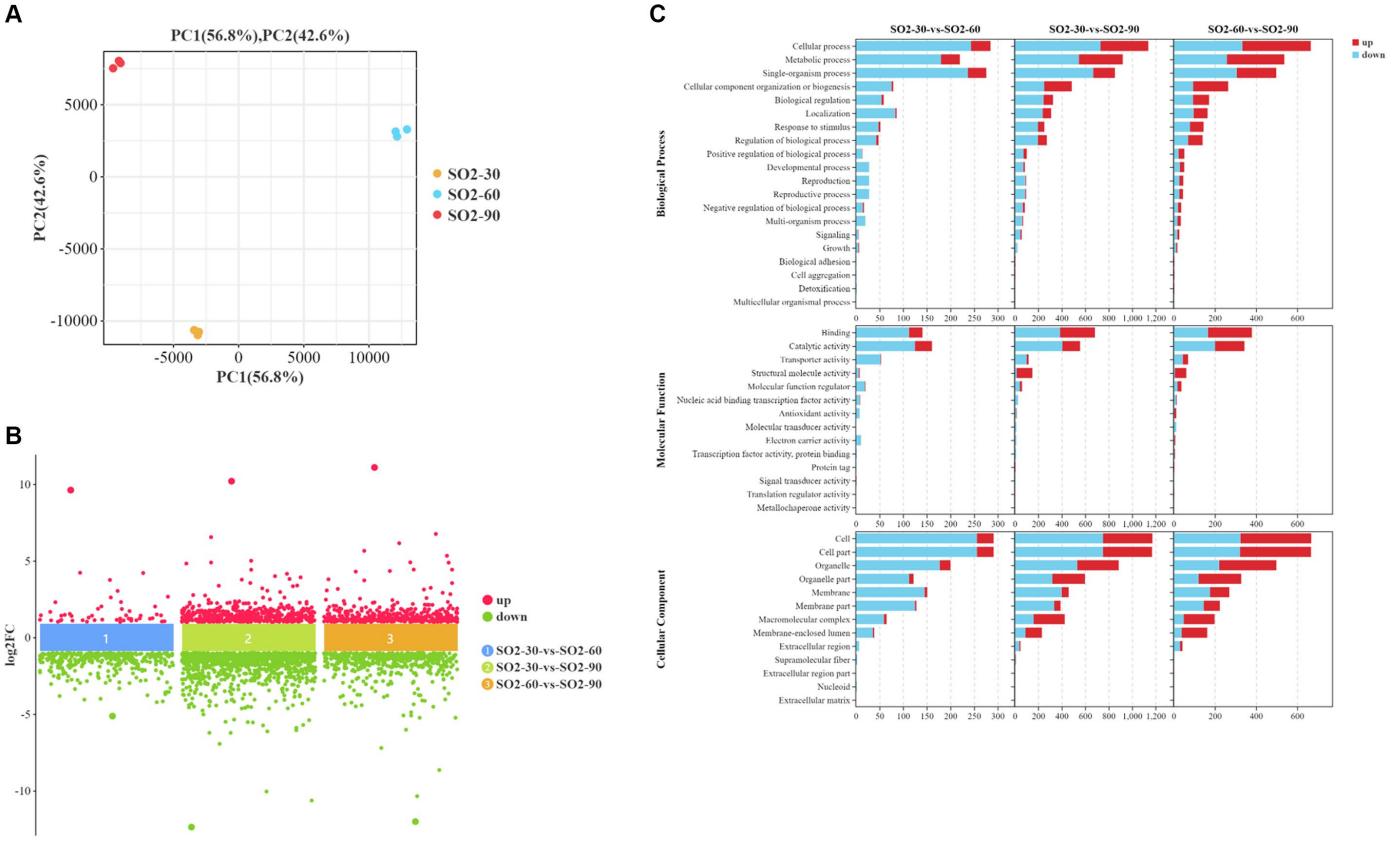
Screening of differentially expressed genes (DEGs) and Gene Ontology (GO) enrichment analysis. **(A)** Principal component analysis (PCA) **(B)** A multi-group difference scatter plot calculates the logarithmic value of the fold difference between the groups (log2FC) as the ordinate and the name of the comparison group as the abscissa. It displays the differential genes of multiple comparison groups simultaneously. The upper part of the abscissa indicates the upregulated genes, and the lower part shows the downregulated genes. **(C)** GO enrichment analysis using secondary classification histogram maps and differential gene transcripts. The differential gene transcripts obtained from the GO database are divided into the following groups: molecular function (MF), cellular component (CC), and biological process (BP). Regarding the included GO terms, the abscissa is the secondary GO term, and the ordinate is the number of differential genes in the term. Red indicates upregulation, and blue indicates downregulation.

**Figure 4:**
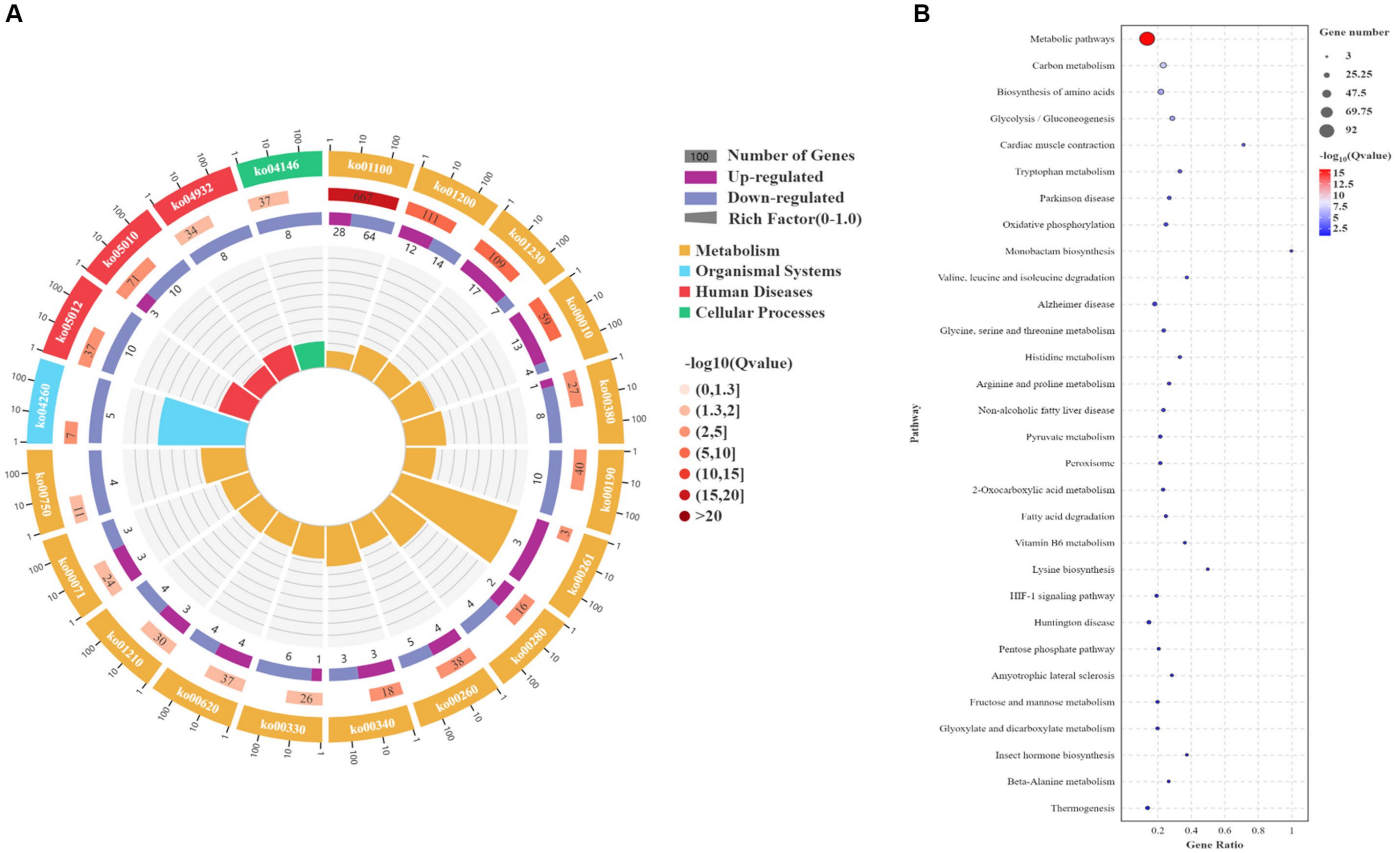
Kyoto Encyclopedia of Genes and Genomes (KEGG) enrichment analysis of differentially expressed genes (DEGs). **(A)** KEGG enrichment circle plot. Moving from the outer to the inner circle, this diagram displays the number of genes enriched in the background gene set, the number of genes enriched in the differential gene set, and the Gene Ratio. **(B)** KEGG enrichment bubble chart. The ordinate represents the pathway, and the abscissa represents the enrichment factor (the number of differential genes in the pathway divided by the total number of genes in the pathway). The size of the dot indicates the quantity of genes (gene number). The color chart represents the *p* value: dark red signifies a low *p* value.

Based on the transcriptomic analysis results, PCA showed reliable repeatability in the three parallel samples and identified 2,766 DEGs, of which 937 were upregulated and 1829 were downregulated (Figures 3A,B). GO and KEGG identified a total of 6,510 GO terms that were enriched in biological process (BP; 34.29%), molecular function (MF; 30.38%), and cellular component [CC; 35.33%] (Figure 3C). According to the results of the BP analysis, with the increase in SO_2_ concentration, the number of upregulated and downregulated DEGs also increased. Metabolic processes related to the synthesis of higher alcohols include pyruvate metabolism that is catabolized into alcohol through the Ehrlich pathway (GO:0006090), glycolysis (GO:0006096), amino acid metabolism (GO:1901605), oxidation–reduction process (GO:0055114), redox coenzyme metabolism (GO:0006733), and oxidative phosphorylation (GO:0006119). The results of the MF analysis relating to the synthesis of higher alcohols included phosphoenolpyruvate carboxykinase (ATP) activity (GO:0004612), aldehyde dehydrogenase (NAD) activity (GO:0004029), pyruvate decarboxylase activity (GO:0004737), and transaminase activity (GO:0008483). KEGG enrichment analysis revealed that the pathway classes with significantly enriched differential genes mainly included metabolic pathways, carbon metabolism, amino acid biosynthesis, glycolysis, and gluconeogenesis (Figures 4A,B). The pathways related to the synthesis of higher alcohols mainly involve carbon metabolism (ko01200), pentose phosphate pathway (ko00030), pyruvate metabolism (ko00620), glycolysis/gluconeogenesis (ko00010), and the valine, leucine, and isoleucine anabolic pathways (ko00290). In addition, the regulation of DEGs examined by RT-qPCR was consistent with that observed using RNA-seq data (Supplementary Figures 2A–D).

We conducted a trend analysis on three groups of gradient samples with varying initial SO_2_ concentrations (30, 60, and 90 mg/L). Clustering was based on the gene expression patterns of the samples (the shape of the expression curve in multiple stages), in order to facilitate the selection of representative gene sets that met specific biological characteristics in the clustering result (Zeng et al., 2019). The overall findings of the trend analysis are shown in Supplementary Figure 3. A total of seven change trends was observed. Based on the change trend observed in the cumulative concentration of higher alcohols (total higher alcohol) and the gene expression function, the following three gene expression trends were analyzed. Twenty-eight genes exhibited a gene expression trend that initially increased and subsequently decreased in relation to the SO_2_ content, including *ADH4, ADH5, GDH2, FBA1, LEU2, TIR1*, and *TPI1*. Eighty-nine genes showed an initial downward and a subsequent upward trend, these included *ALD6* and *SOL4*. *GPD1* and *PCK1* showed a continuous downward trend.

### 3.3 Metabolomic analysis

**Figure 5:**
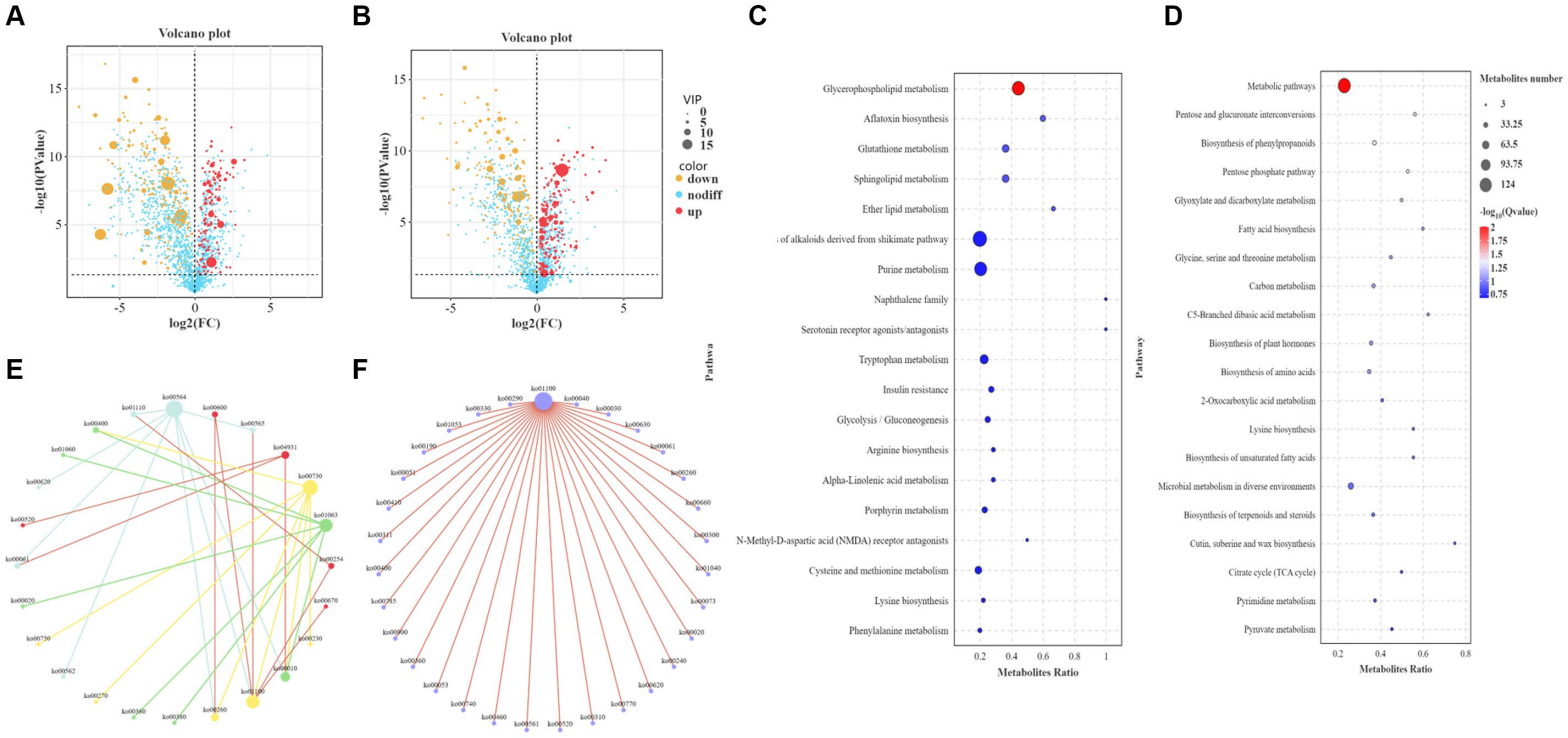
Functional analysis of differential metabolites. **(A)** Volcano plot of differential metabolites in the positive ion mode (POS). The abscissa is the log2 value of the difference in metabolite abundance for each control group, the ordinate is the log transformed *p* value (−log10), and the dotted line (perpendicular to the *Y*-axis) is the threshold of the *p* value for differential metabolite screening. Red dots represent differential metabolites with VIP ≥ 1 and *p* ≤ 0.05, whose expression is upregulated; yellow dots represent differential metabolites with VIP ≥ 1 and *p* ≤ 0.05, whose expression is downregulated. The larger the point, the greater the VIP value of the metabolite. **(B)** Volcano plot of differential metabolites in negative ion mode (NEG). **(C)** Bubble plot of KEGG enrichment in POS. The ordinate is the pathway, and the abscissa is the enrichment factor (the number of differential metabolites in the pathway divided by the total number of metabolites in the pathway). The larger the value, the more significant the enrichment. **(D)** Bubble plot of KEGG enrichment in NEG. **(E)** KEGG pathway interaction network diagram in POS. Mutually related pathways are depicted using identical colors. **(F)** Interaction network diagram of KEGG pathways in NEG.

The metabolites identified by metabolomics and related to the synthesis of higher alcohols are shown in Supplementary Table S3. Metabolomic analysis identified 946 differential metabolites in POS mode, of which 416 were upregulated and 530 were downregulated (Figure 5A). In total, 943 differential metabolites were identified in NEG mode, of which 485 were upregulated and 458 were downregulated (Figure 5B). KEGG analysis was performed on different metabolites in the POS and NEG modes. The results showed that the different metabolic pathways in POS mode (Figure 5C) mainly included glycerophospholipid metabolism (ko00564), glutathione metabolism (ko00480), and the biosynthesis of alkaloids derived from the shikimate pathway (ko01063). The main differential metabolites were acetylcholine (C01996), acetyl coenzyme A (C00024), and glutathione (C00051). In NEG mode (Figure 5D), the affected metabolic pathways included the pentose phosphate pathway (ko00030), amino acid metabolism (ko01230), metabolic pathway (ko01100), and carbon metabolism (ko01200). The main differential metabolites include S-adenosylmethionine (C00019), pyruvate (C00022), α-ketoglutarate (C00026), glycerol (C00116), threonine (C00188), isoleucine (C00407), and glutamine acid (C00302).

In addition, the core pathways in POS mode (Figure 5E) were the glycerophospholipid metabolism pathway, whose main related pathways are ether lipid metabolism and glycolysis/gluconeogenesis, and metabolic pathways; the glutathione metabolism pathway, whose main related pathways are arginine biosynthesis, cysteine and methionine metabolism; biosynthesis of alkaloids derived from the shikimate pathway, whose main related pathways are phenylalanine metabolism, citrate cycle, phenylalanine, tyrosine and tryptophan biosynthesis; thiamine metabolism, whose main related pathways are glycine, serine and threonine metabolism, cysteine and methionine metabolism. The core pathway in NEG mode (Figure 5F) was the metabolic pathway, with its main related pathways being the pentose and glucuronate interconversions, the pentose phosphate pathway, fatty acid biosynthesis, glycine, serine, and threonine metabolism, glyoxylate and dicarboxylate metabolism, and valine, leucine, and isoleucine biosynthesis.

### 3.4 Transcriptome and metabolome association analysis

**Figure 6:**
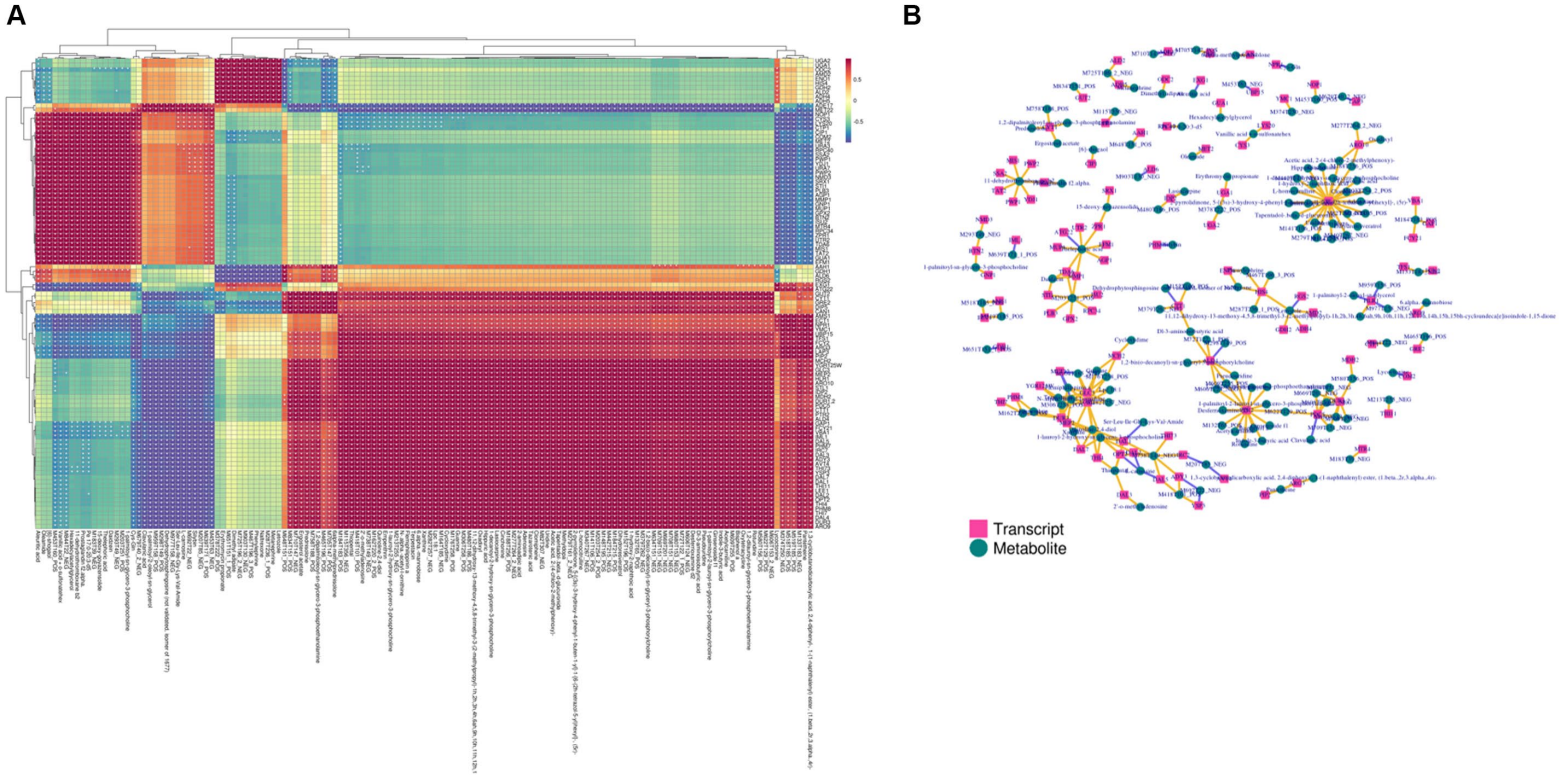
Transcriptome and metabolome association analysis. **(A)** Heat map of the correlation between gene expression and metabolite abundance. **(B)** Network diagram of the correlation between gene expression and metabolite abundance. The yellow line indicates a positive correlation, and the purple line indicates a negative correlation.

The DEGs obtained from transcriptomics and differential metabolites obtained from metabolomics were subjected to KEGG pathway analysis. The most common metabolic pathways include glycolysis/gluconeogenesis, carbon metabolism, amino acid biosynthesis, tryptophan metabolism, and the TCA cycle. The O2PLS model was used to perform a correlation analysis on the two sets of scientific data. The O2PLS load diagram (Supplementary Figures 4A–C) of the joint part (the part that is highly related to the transcriptome and metabolome) and the two sets of scientific correlation load diagrams (Supplementary Figures 5A–C) were used to obtain a combined analysis of the correlated gene and metabolite sets. The correlation between the top 250 DEGs and differential metabolites with correlation coefficients and absolute values greater than 0.5 is represented by a heat map (Figure 6A) and a network diagram (Figure 6B). In the correlation analysis, differential metabolites related to the synthesis of higher alcohols include pyruvate, valine, leucine, isoleucine, tyrosol, and glycerol, and related genes include *CDC19* (pyruvate kinase), *CYB2* (L-lactate dehydrogenase, cytochrome b2), *ENO2* (phosphopyruvate hydratase), *ALD4* (mitochondrial aldehyde dehydrogenase), *ALD6* (cytosolic aldehyde dehydrogenase), *ARO10* (phenylpyruvate decarboxylase), *PDCs* (pyruvate decarboxylase), *ADH4* (alcohol dehydrogenase isoenzyme type IV), *ADH5* (Alcohol dehydrogenase isoenzyme V), *BDH2* (alcohol dehydrogenase), *TPI1* (triose phosphate isomerase), *HXT2* (glucose transporter), *FBA1* (fructose 1,6-bisphosphate aldolase), and *GDH2* (NAD^+^-dependent glutamate dehydrogenase).

### 3.5 Effect of single gene knockout on the production of higher alcohols

**Figure 7:**
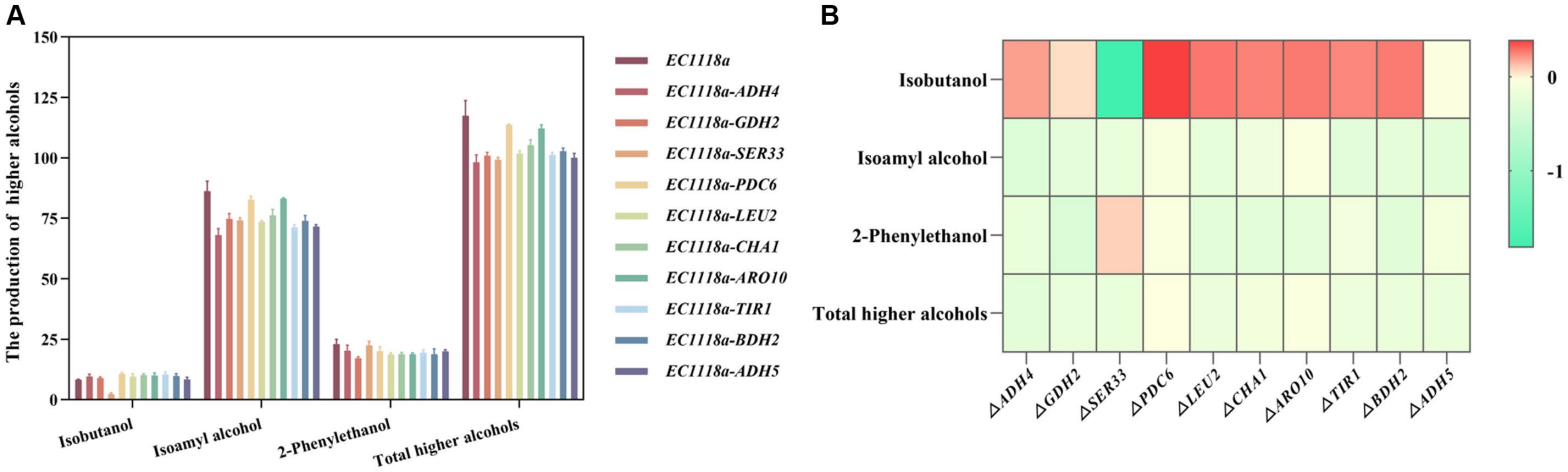
Histogram and heat map depicting higher alcohol production in single gene deletion strains. **(A)** The production of isobutanol, isoamyl alcohol, 2-phenylethanol, and the cumulative concentration of higher alcohols (total higher alcohols) after deleting the *ADH4, GDH2, SER33, PDC6, LEU2, CHA1, ARO10, TIR1, BDH2*, and *ADH5* gene strains. **(B)** The log2 value of the ratio of higher alcohols between each recombinant strain and the parent strain was used to construct a heat map. Red indicates an increase in the metabolite content, and green indicates a decrease in the metabolite content. Color intensity represents the amount of upward or downward adjustment. The darker the color, the greater the proportional increase or decrease in the metabolite. The scale bar on the right depicts the maximum or minimum rate of change. The data are the mean ± SD from three biological replicates.

First, DEGs and differential metabolites were obtained through single-omics analyses such as PCA and differential enrichment analysis. Based on the changing characteristics of gene expression at different omics levels, DEGs and different metabolites were subjected to O2PLS analysis and various correlation analyses to explore the potential molecular connections between different omics levels. Starting from the target differential metabolites related to the anabolism of higher alcohols, the associated differential genes were obtained and combined using existing research and trend analysis. Ten target DEGs (*ADH4*, *GDH2*, *SER33*, *PDC6*, *LEU2*, *CHA1*, *ARO10*, *TIR1*, *BDH2*, and *ADH5*) were chosen for the knockout experiments. Single-gene knockout strains were constructed to explore the effect of their knockout on the production of higher alcohols. The total alcohol synthesis in each knockout strain was lower than that in the original strain (Figures 7A,B). Among them, the EC1118a*-ADH4*, EC1118a*-SER33*, and EC1118a*-ADH5* knockout strains showed a more significant reduction effect, with the total higher alcohol content reduced by 16.479, 15.620, and 14.771%, respectively. For isobutanol, only the EC1118a*-SER33* knockout strain showed a decreasing trend and the effect was significant with a reduction of 71.346%; whereas for isoamyl alcohol, the EC1118a*-ADH4* and EC1118a*-TIR1* knockout strains showed a significant reduction of 21.003 and 17.334%, respectively. Regarding 2-phenylethanol, the effects of EC1118a*-GDH2*, EC1118a*-CHA1*, and EC1118a*-ARO10* knockout were more significant, with reductions of 25.198, 18.234, and 18.016%, respectively.

## 4 Discussion

Once entering the cell, SO_2_ can bind to proteins, coenzymes (NAD^+^ and FAD^+^), vitamins, and various metabolites (acetaldehyde, glucose, oxaloacetate, and α-ketoglutarate); thereby, preventing them from being further catabolized as substrates of metabolic pathways, leading to the appearance and prolongation of the lag phase (Herrero et al., 2003; Machín et al., 2004). This aligns with our experimental findings, where a significant increase in SO_2_ inhibited strain growth and prolonged the lag period. The fluctuating trend observed in higher alcohol production, initially increasing before subsequently decreasing, is consistent with the previously reported findings (Bloem et al., 2016; Ochando et al., 2020). The appropriate levels of SO_2_ may stimulate the production of pyruvate, a precursor to higher alcohol carbon metabolism. This process may protect α-acetolactate (a precursor to higher alcohols) from oxidative decarboxylation, a process in which diacetyl (a competitive precursor substance for the sugar metabolism of higher alcohols) is formed (Ochando et al., 2020). This process is conducive for the generation of higher alcohols. In contrast, excessive SO_2_ concentrations inhibit strain growth and cause a lag phase, affecting the anabolism of higher alcohols and other substances.

Metabolic pathways related to the synthesis of higher alcohols that are enriched in transcriptome DEGs mainly include carbon and pyruvate metabolism. Results of the trend analysis demonstrated that compared with SO_2_ concentrations of 30 mg/L, *ADH4* gene expression in the 60 mg/L and 90 mg/L SO2 experimental groups was upregulated by 2.31-fold and downregulated by 1.95-fold. The expression of *ADH5* was upregulated by 1.5-fold and downregulated by 0.68-fold. During higher alcohol synthesis, *ADH4* and *ADH5* encode the alcohol dehydrogenases 1 (Adh1) and 2 (Adh2), respectively. Alcohol dehydrogenase reduces aldehydes to their corresponding alcohols (Bae et al., 2016), and high expression levels of *ADH4* and *ADH5* increase alcohol dehydrogenase activity, which is beneficial for the synthesis of higher alcohols. The expression of the *ALD6* gene was downregulated by 1.91-fold and upregulated by 0.22-fold. The *ALD6* gene encodes aldehyde dehydrogenase, which catalyzes the dehydrogenation and oxidation of aldehydes to generate their corresponding acids. Downregulation of this gene leads to an increase in alcohol content (Zheng et al., 2020). *Saccharomyces cerevisiae* can use either the shikimate pathway for the *de novo* synthesis of phenylalanine, or it can convert L-phenylalanine under the catalysis of two aminotransferases (Shen et al., 2016). In the anabolic pathway, pyruvate can be used to synthesize isoleucine, leucine, valine, and α-keto acids through the branched-chain amino acid anabolic pathway, thereby generating higher alcohols. In the tricarboxylic acid cycle, the addition reaction of oxaloacetate and amino groups produces aspartic acid. The enzymatic reaction of aspartic acid produces threonine. Moreover, threonine and glycine can be interconverted through the action of threonine aldolase to generate α-ketobutyrate (Nishimura et al., 2018). Metabolomic analyses revealed that core metabolic pathways and differential metabolites correlate with the anabolism of higher alcohols, this indirectly reflects the potential of these metabolites to influence higher alcohol production.

*GDP1* (NAD-dependent glycerol-3-phosphate dehydrogenase) plays a key role in the synthesis of glycerol and intracellular glycerol accumulation in response to high osmotic stress (Hubmann et al., 2011). The expression of *GPD1* was downregulated by 1.11- and 0.57-fold, at 60 and 90 mg/L SO_2_, respectively. Low expression of *GPD1* leads to a reduction in the flow of metabolites of carbon metabolism to glycerol, which may also indicate an increase in the flow of these metabolites to higher alcohols, thereby facilitating their synthesis. At high SO_2_ levels, yeast must reduce cellular stress and “consume” sulfite, resulting in a higher flux through the pentose phosphate pathway (Ochando et al., 2020). This results in a greater *de novo* anabolic flux of phenylpyruvic acid and a higher accumulation of phenylethyl alcohol and its corresponding ester [phenylethyl acetate] (Cadière et al., 2011). In the present study, the high yield of phenylethanol at high SO_2_ content supports this hypothesis. *P*yruvate kinase (*CDC19*) catalyzes the final step of glycolysis, converting phosphoenolpyruvate into pyruvate; triose phosphate isomerase (*TPI1*) catalyzes the interconversion of dihydroxyacetone phosphate and glyceraldehyde 3-phosphate, intermediates in glycolysis that lead to pyruvate. The expression of *CDC19* was increased by 1.16- and 0.24-fold, and that of *TPI1* by 1.28- and 0.48-fold, by 60 and 90 mg/L SO_2_, respectively, which may increase pyruvate concentration.

It is speculated that SO_2_ acts indirectly as a transcriptional regulator, and the addition of SO_2_ may induce the production of pyruvate, promote the secretion of pyruvate, and protect α-acetolactate from oxidative decarboxylation to diacetyl. The availability of carbon metabolic precursors in the synthesis of higher alcohols such as pyruvate and α-acetolactate and the accessibility of redox cofactors involved in metabolic processes facilitate the synthesis of volatile substances such as higher alcohols (Herrero et al., 2003; Bloem et al., 2016). Further experiments are needed to verify the dynamic analysis of higher alcohol carbon metabolism precursors such as pyruvate and α-acetolactate during the fermentation process, as well as monitoring the impact of SO_2_ addition on redox homeostasis and redox cofactors.

In *S. cerevisiae*, α-keto acids are converted into aldehydes within the cytoplasm through α-keto acid decarboxylase encoded by genes such as *PDCs*, *ARO10*, and *THI3*. These aldehydes are then synthesized into higher alcohols via the catalysis of alcohol dehydrogenases such as *ADHs*. Additionally, aldehydes can also be dehydrogenated and oxidized to form their corresponding acids via aldehyde dehydrogenases encoded by *ALDs.* Therefore, higher alcohol production can be reduced, by reducing the decarboxylase and alcohol dehydrogenase levels while increasing the activity of aldehyde dehydrogenase (Dzialo et al., 2017). The *SER33* gene (3-phosphoglycerate dehydrogenase and alpha-ketoglutarate reductase) catalyzes the first step in the biosynthesis of serine and glycine (Kobayashi et al., 2022), whereas the *TIR1* gene (cell wall mannoprotein) is related to the maintenance of the plasma membrane and cell wall (López-Malo et al., 2015). The role of the *SER33* and *TIR1* genes in the regulation of higher alcohol metabolism in the EC1118a strain is currently unclear. Further studies are required to fully understand their role in the synthesis of higher alcohols. The *GDH2* gene encodes NAD^+^-dependent glutamate dehydrogenase, which degrades glutamate into ammonia and α-ketoglutarate, regulating the metabolism of branched-chain amino acids. The knockout of this gene affects the formation of 2-phenylethanol, and the results of Wang et al. (2021) support this conclusion.

## 5 Conclusion

This study reports the effect of the initial concentration of SO_2_ on higher alcohol metabolism in yeast. As the initial SO_2_ content increased, the total higher alcohol production initially increased and then decreased. Transcriptome and metabolome correlation analyses showed that the addition of SO_2_ affected carbon metabolism, amino acid metabolism, pyruvate metabolism, glycolysis/gluconeogenesis, the pentose phosphate pathway, and other metabolic pathways related to the synthesis of higher alcohols. SO_2_ affects the availability of carbon metabolism precursors and the accessibility of redox cofactors, which indirectly affects the synthesis of higher alcohols. Combining correlation and trend analyses of DEGs and metabolites, we screened 10 genes to construct single-gene knockout strains and found that the total alcohol content of the modified strains was lower than that of the original strains. *ADH4*, *SER33*, and *GDH2* genes were found to be significant in the production of higher alcohols in *S. cerevisiae*. These results lay the foundation for studying the mechanism of initial SO_2_ addition in the synthesis of higher alcohols in *S. cerevisiae*. The discovery of new target genes will aid in the construction of new strains with lower alcohol production.

## Data availability statement

The data presented in the study are deposited in the SRA repository, accession number PRJNA1104300.

## Author contributions

YuL: Data curation, Investigation, Methodology, Validation, Writing – original draft, Writing – review & editing. NZ: Conceptualization, Investigation, Writing – original draft, Writing – review & editing. YoL: Data curation, Methodology, Writing – original draft, Writing – review & editing. YG: Data curation, Methodology, Writing – review & editing, Writing – original draft. HL: Conceptualization, Formal analysis, Writing – original draft, Writing – review & editing. CZ: Conceptualization, Formal analysis, Writing – original draft, Writing – review & editing. WM: Conceptualization, Formal analysis, Supervision, Writing – original draft, Writing – review & editing. WQ: Conceptualization, Formal analysis, Supervision, Writing – original draft, Writing – review & editing.

## Footnotes

^1^ http://ftp.ensemblgenomes.org/pub/fungi/release-47/gtf/saccharomyces_cerevisiae/

^2^ http://www.r-project.org/

^3^ http://www.geneontology.org/

^4^ http://www.genome.jp/kegg/
