# Supplementary material for: Transcriptomic and metabolomic correlation analysis: effect of initial SO2 addition on higher alcohol synthesis in Saccharomyces cerevisiae and identification of key regulatory genes
Source: Front Microbiol. 2024 May 13;15:1394880. doi: 10.3389/fmicb.2024.1394880 (PMC11128613; doi:10.3389/fmicb.2024.1394880)
Supplement: Supplementary file 1 [file Data_Sheet_1.docx]

Supplementary Material

Table S1. Plasmids and Primers.

| **Plasmids and Primers** | **Description** | **Source** |
| --- | --- | --- |
| pUG6 | Ap^r^, containing *loxP-KanMX-loxP* cassette | This lab |
| pSH69 | Hyb^r^, Cre expression vector | This lab |
| ADH4-U-F | GAGGCTGCTGATTTGAAATTGCGTTTTGTG | This study |
| ADH4-U-R | ctgcagcgtacgaagcttcTTTTCTTATTTGACTATTAG | This study |
| ADH4-K-F | CTAATAGTCAAATAAGAAAAgaagcttcgtacgctgcag | This study |
| ADH4-K-R | TTTATGAGTTCGTTCGATTTTTgcataggccactagtgga | This study |
| ADH4-D-F | tccactagtggcctatgcAAAAATCGAACGAACTCATAAA | This study |
| ADH4-D-R | CGCATGTGAATGACACACGAAAGTAATCGGA | This study |
| GDH2-U-F | CGCAAGTAATACTTACAGCACTGTAACCATAGGT | This study |
| GDH2-U-R | ctgcagcgtacgaagcttcTCTTTATTCTTTTTATTGTTGTG | This study |
| GDH2-K-F | CACAACAATAAAAAGAATAAAGAgaagcttcgtacgctgcag | This study |
| GDH2-K-R | CAAAACAATTTTATTGAAGCgcataggccactagtgga | This study |
| GDH2-D-F | tccactagtggcctatgcGCTTCAATAAAATTGTTTTG | This study |
| GDH2-D-R | TGAAATTCAGGTTCTTGCGACGGCTTGA | This study |
| SER33-U-F | CAATGGGGTTCGCAAACATGGTTAATGAAGGCT | This study |
| SER33-K-F | TTTCGGGTATTTCCTCCCTAACgaagcttcgtacgctgca | This study |
| SER33-U-R | tgcagcgtacgaagcttcGTTAGGGAGGAAATACCCGAAA | This study |
| SER33-D-F | tccactagtggcctatgcTTACTCAGATAAATAAAAATATATGT | This study |
| SER33-K-R | ACATATATTTTTATTTATCTGAGTAAgcataggccactagtgga | This study |
| SER33-D-R | GGTATCGTCATCATACAATGCCTCAATATCCGGCAGT | This study |
| PDC6-U-F | ATGTCCATTGGAATATGCAGATCGGCTGTGG | This study |
| PDC6-K-F | CAAAAACATATTGCCAACAAAgaagcttcgtacgctgca | This study |
| PDC6-U-R | tgcagcgtacgaagcttcTTTGTTGGCAATATGTTTTTG | This study |
| PDC6-D-F | tccactagtggcctatgcGCCATTAGTAGTGTACTC | This study |
| PDC6-K-R | GAGTACACTACTAATGGCgcataggccactagtgga | This study |
| PDC6-D-R | CAAACGACGGGAAACAAGGAAGGATGCAGAAT | This study |
| LEU2-U-F | ATTGTCCTGTACTTCCTTGTTCATGTGTGTTC | This study |
| LEU2-K-F | ATATATTTCAAGGATATACCATTCTAgaagcttcgtacgctgca | This study |
| LEU2-U-R | tgcagcgtacgaagcttcTAGAATGGTATATCCTTGAAATATAT | This study |
| LEU2-D-F | tccactagtggcctatgcAAAGATTCTCTTTTTTTATGATATTTG | This study |
| LEU2-K-R | CAAATATCATAAAAAAAGAGAATCTTTgcataggccactagtgga | This study |
| LEU2-D-R | AGCCCATTCTTCCATCAGATTTGGTATTGGT | This study |
| CHA1-U-F | TCTCCGTGATATCCTCTAGGGCTTGGGTTGCC | This study |
| CHA1-K-F | GAAAATTAACCAGCGAGgaagcttcgtacgctgca | This study |
| CHA1-U-R | tgcagcgtacgaagcttcCTCGCTGGTTAATTTTC | This study |
| CHA1-D-F | tccactagtggcctatgcAAATCCAATAACTTTTTCGTTGAAGC | This study |
| CHA1-K-R | GCTTCAACGAAAAAGTTATTGGATTTgcataggccactagtgga | This study |
| CHA1-D-R | GATGAAAGAGCTGCAAATGTCGGCAAAAT | This study |
| ARO10-U-F | GCAGGATTGAAAGCGTACAACAACGTCTTAGCG | This study |
| ARO10-K-F | GATAACAAAGAAACTCCCTTAAGCgaagcttcgtacgctgca | This study |
| ARO10-U-R | tgcagcgtacgaagcttcGCTTAAGGGAGTTTCTTTGTTATC | This study |
| ARO10-D-F | tccactagtggcctatgcAAACTTGTGGGCG | This study |
| ARO10-K-R | CGCCCACAAGTTTgcataggccactagtgga | This study |
| ARO10-D-R | GCTACCAACTTGCTTACCAACTCTTCTTGGACCGT | This study |
| TIR1-U-F | GACCCGTTTTAGTGCCTGTCCCTT | This study |
| TIR1-K-F | CAACAAGTACTACAATAATTAAAAgaagcttcgtacgctgca | This study |
| TIR1-U-R | tgcagcgtacgaagcttcTTTTAATTATTGTAGTACTTGTTG | This study |
| TIR1-D-F | tccactagtggcctatgcGTTATTGGTTTTTAATAAT | This study |
| TIR1-K-R | ATTATTAAAAACCAATAACgcataggccactagtgga | This study |
| TIR1-D-R | TGCCACCCCATTTCTTTAACGCCAGT | This study |
| BDH2-U-F | ATGGTGGCTGACGCAATGCTTGAC | This study |
| BDH2-K-F | TTCATTGAACATATTTCAGAgaagcttcgtacgctgca | This study |
| BDH2-U-R | tgcagcgtacgaagcttcTCTGAAATATGTTCAATGAA | This study |
| BDH2-D-F | tccactagtggcctatgcTTGTGATTGAGTACTCAC | This study |
| BDH2-K-R | GTGAGTACTCAATCACAAgcataggccactagtgga | This study |
| BDH2-D-R | CCAAGTACTCGTGAAGATCCGAGCCACAAAT | This study |
| ADH5-U-F | CAAGTAGTGTCTTCATGACGGATTCATAGTCTATCCAAGC | This study |
| ADH5-U-R | tgcagcgtacgaagcttcGATGCTTTGATTTTGTAGATATGTAGTTAA | This study |
| ADH5-K-F | TTAACTACATATCTACAAAATCAAAGCATCgaagcttcgtacgctgca | This study |
| ADH5-K-R | ATTCATCAAATTCGTTACAAAAGAgcataggccactagtgga | This study |
| ADH5-D-F | tccactagtggcctatgcTCTTTTGTAACGAATTTGATGAAT | This study |
| ADH5-D-R | TCCTTCTGGGCAATTCATCCTCGGTTTTTTGATAAATAGTCC | This study |
| For PCR verifcation |  | This study |
| ADH4-S-F | GAGGCTGCTGATTT | This study |
| ADH4-X-R | CCGTTGTGTTTCGAGGTTAT | This study |
| GDH2-S-F | AAACATCGATCAACGATACG | This study |
| GDH2-X-R | GGGTGTAACTCGGAATATCTG | This study |
| SER33-S-F | TGGTGTCCTTCAAACAGAGTT | This study |
| SER33-X-R | CGGCAAGTGCATACTTCACA | This study |
| PDC6-S-F | ACTTGGCAATAGATGAGCATT | This study |
| PDC6-X-R | CTTTGCAATACTGCTAACAATGGA | This study |
| LEU2-S-F | GTGTAGAATTGCAGATTCCCTT | This study |
| LEU2-X-R | TGGCACTAAGGGATATCGCA | This study |
| CHA1-S-F | TCCAATGACTCTTGATCTGTTG | This study |
| CHA1-X-R | CAAACGAGGGGCTTTACTGTA | This study |
| ARO10-S-F | ATAATTTGGCGAACCCCTTTT | This study |
| ARO10-X-R | CCTCTCTAGTGGTGTCCAAGA | This study |
| TIR1-S-F | GTGAACCGTTTCCATCTAACT | This study |
| TIR1-X-R | CCCTTTTGTGGATTCGTATGTC | This study |
| BDH2-S-F | CTTCAACGACTGCATACAGG | This study |
| BDH2-X-R | TTGTCACTTTAGGACCAACCT | This study |
| ADH5-S-F | CGAAGTTGCGTCGAAGATGA | This study |
| ADH5-X-R | CGCCGATTAAAGCGTACTCAT | This study |
| KAN-X-F | tctgaaacatggcaaaggtagc | This study |
| KAN-S-R | gcaagatcctggtatcggtc | This study |

Table S2. qPCR Primers.

| Primers | Description | Source |
| --- | --- | --- |
| Q-GPD1-F | CTTCTGGTAAGGACGCCTGG | This study |
| Q-GPD1-R | CATGTCCGGCAGGTTCTTCA | This study |
| Q-ADH5-F | TCTTGCTGAAGTTGCCCCAA | This study |
| Q-ADH5-R | CCGCATGCACCGGATATAGT | This study |
| Q-TPI1-F | CGTTAGCGGAACCACCGTAT | This study |
| Q-TPI1-R | GTACCGGTTTGGCTGCTACT | This study |
| Q-ALD6-F | CGAGCAACCAACCGGTCTAT | This study |
| Q-ALD6-R | CGAGCAACCAACCGGTCTAT | This study |

Table S3. Mainly associated differential metabolites.

| Metabolite | KEGG ID | Sub Class |
| --- | --- | --- |
| valine | C16436 | Carboxylic acids and derivatives |
| Leucine | C00123 | Carboxylic acids and derivatives |
| Aspartic acid | C00049 | Carboxylic acids and derivatives |
| Isoleucine | C00407 | Carboxylic acids and derivatives |
| Glutamine | - | Carboxylic acids and derivatives |
| Glutamic acid | C00302 | Carboxylic acids and derivatives |
| Pyruvate | C00022 | Keto acids and derivatives |
| alpha-ketoglutarate | C00026 | Keto acids and derivatives |
| NAD+ | C00003 | - |
| NADH | C00004 | - |
| Glycerol | C00116 | Organooxygen compounds |
| Fructose 1,6-diphosphate | C00354 | Organooxygen compounds |
| Shikimate | C00493 | Organooxygen compounds |
| Acetylcholine | C01996 | Organooxygen compounds |
| Vanillic acid | C06672 | Benzene and substituted derivatives |


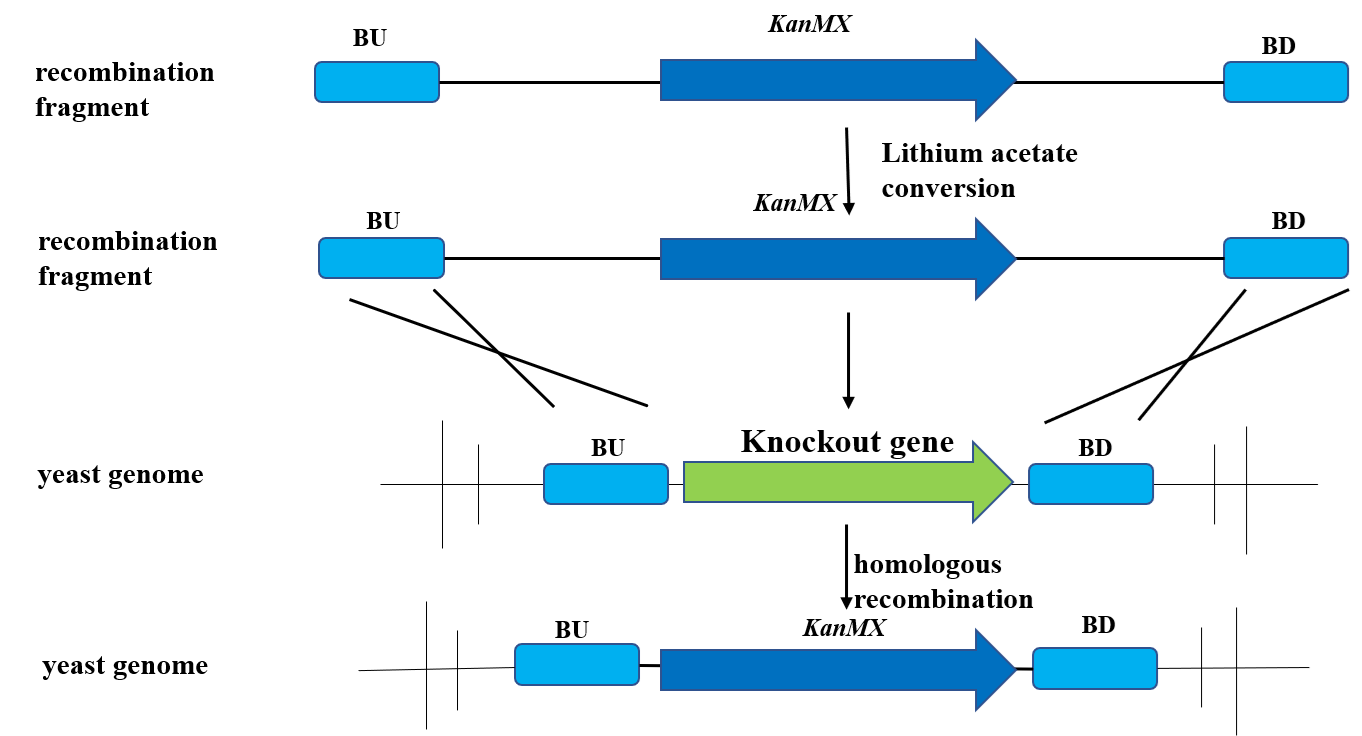


**Supplementary Figure 1.** Principle of homologous recombination for gene knockout in yeast genome.


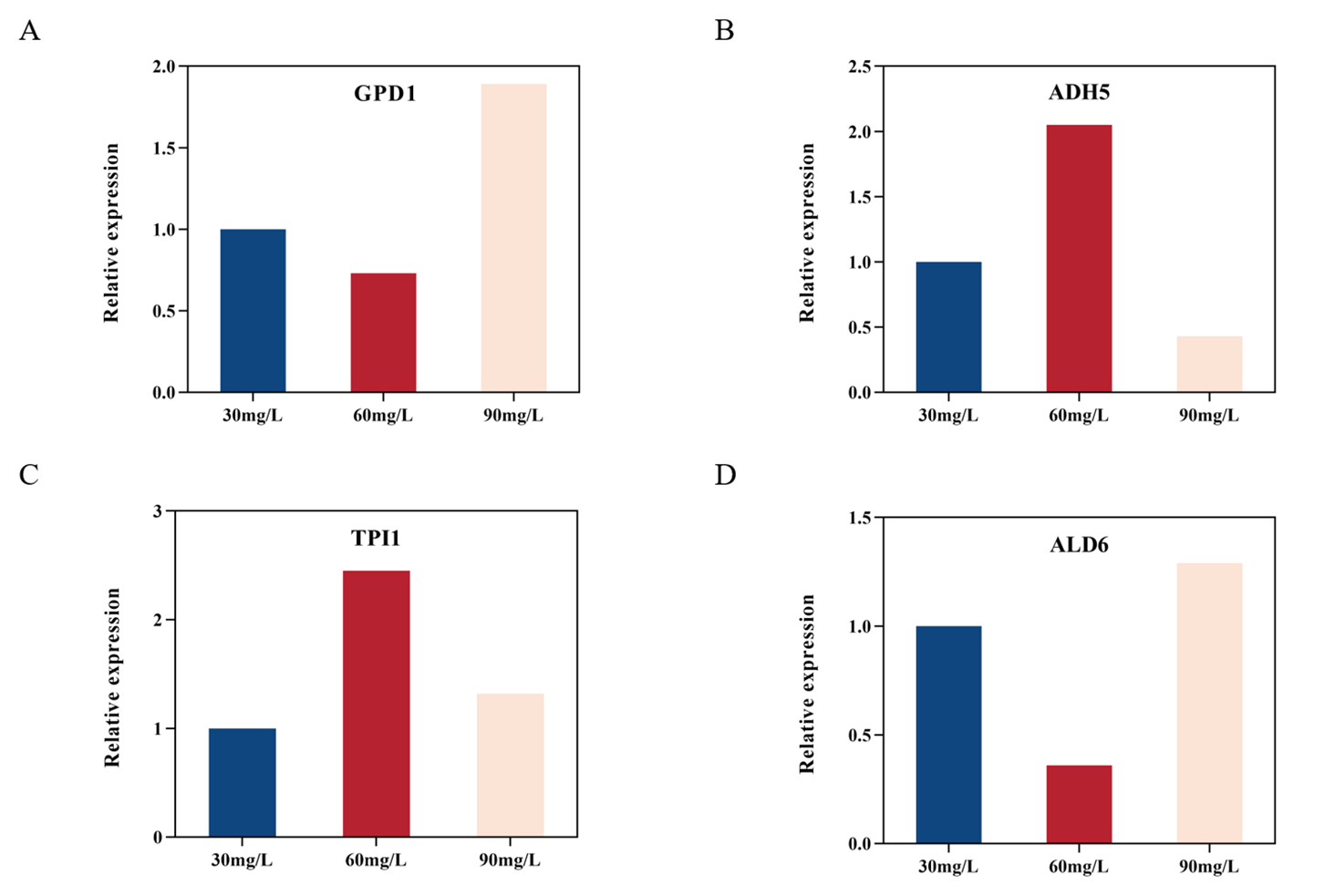


**Supplementary Figure 2.** qPCR experimental results (taking GPD1, ADH5, TPI1, ALD6 as examples).


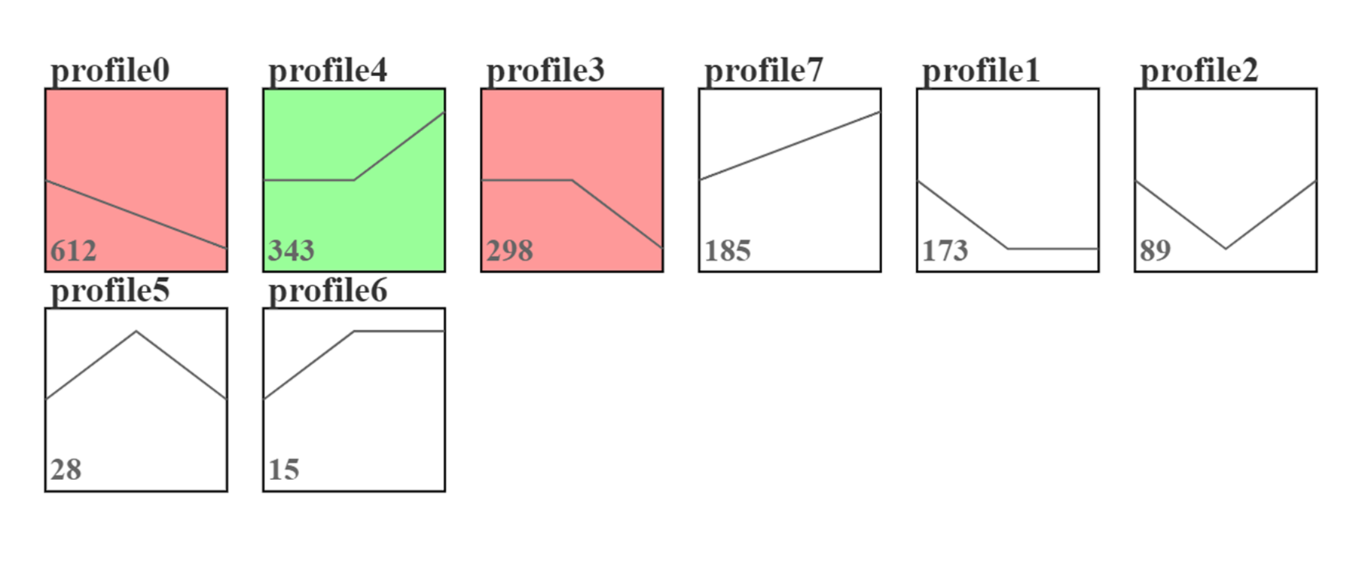


**Supplementary Figure 3.** Overall trend analysis of DEGs.


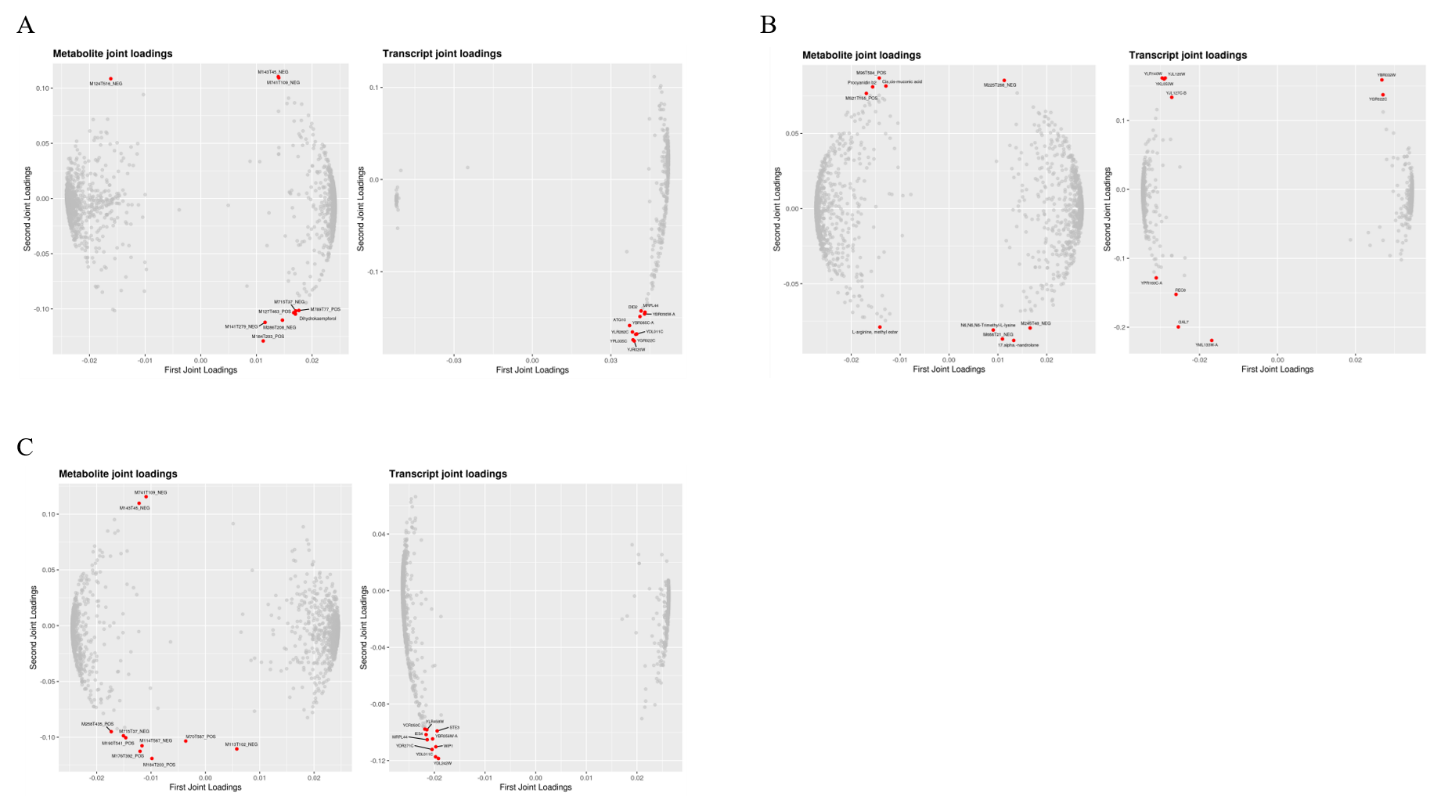


**Supplementary Figure 4.** O2PLS load diagram. Metabolome data is on the left and transcriptome data is on the right. Each dot represents a gene or a metabolite. The abscissa is the one-dimensional coordinate of the joint part, and the ordinate is the two-dimensional coordinate of the joint part. The greater the absolute value of an element in a coordinate, the greater the correlation between that element and another omics. (A) 30/60mg/L comparison. (B)60/90mg/L comparison. (C) 30/90mg/L comparison.


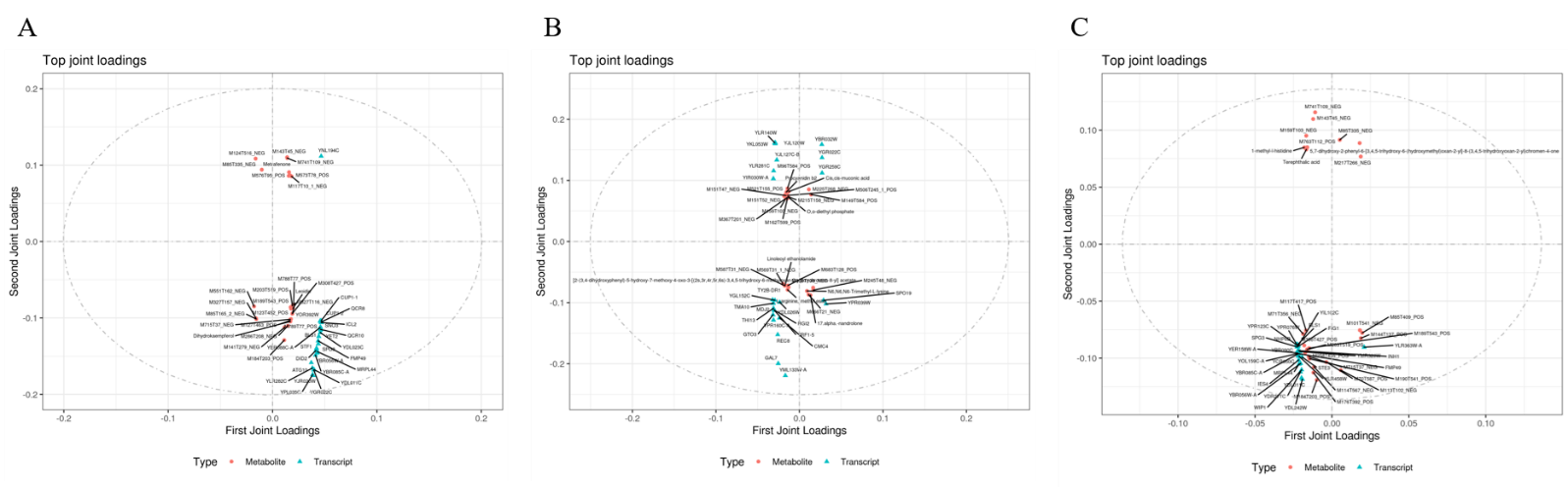


**Supplementary Figure 5.** Two sets of scientific correlation loading diagrams. (A) 30/60mg/L comparison. (B)60/90mg/L comparison. (C) 30/90mg/L comparison.
